# Projection of climate change impacts on extreme temperature and precipitation in Central Poland

**DOI:** 10.1038/s41598-023-46199-5

**Published:** 2023-10-31

**Authors:** Babak Ghazi, Rajmund Przybylak, Aleksandra Pospieszyńska

**Affiliations:** 1 Department of Meteorology and Climatology, Faculty of Earth Sciences and Spatial Management, Nicolaus Copernicus University, Toruń, Poland; 2 Centre for Climate Change Research, Nicolaus Copernicus University, Toruń, Poland

**Keywords:** Climate-change impacts, Projection and prediction

## Abstract

Climate change is exacerbating the risk of the occurrence of extreme weather. This study has projected the change in mean and extreme climate conditions in Central Poland during near-future (2026–2050), mid-term (2051–2075), and far-future (2076–2100) periods under two climate-change scenarios in six General Circulation Models (GCMs) from Coupled Model Intercomparison Project Phase 6 (CMIP6). The results showed that, compared to the historical reference period (1990–2014), Central Poland will experience an increase in temperature and precipitation by the end of the twenty-first century. It is expected that the mean annual temperature and mean annual precipitation totals will increase by 1–4.8 °C and 2–7.5%, respectively. Furthermore, it is projected that the average number of hot, very hot days and extremely hot days (Tmax > 25 °C, > 30 °C, and > 35 °C), tropical nights (Tmin > 20 °C), and extremely high daily precipitation (> 10 mm, > 20 mm and > 30 mm) will also increase, while the average number of slight frost days (Tmin < 0 °C), and frost and severe frost days (Tmax < 0 °C, Tmax <  − 10 °C) will decline on average by the end of the twenty-first century. Therefore, it is essential for policymakers to take some appropriate measurements and strategies in advance to strengthen resilience to extreme climate events.

## Introduction

Climate change is one of the most substantial global issues of the contemporary Anthropocene Era. Today, researchers believe that there is clear evidence that anthropic activities have been the main cause of global warming, primarily due to fossil fuel burning leading to increasing greenhouse gas emissions^1^. Thus, scientists have called the contemporary era “Anthropocene Era”. The Anthropocene Working Group agreed that the Anthropocene began in the 1950s when the Great Acceleration, the most dramatic rise in human activity since the Industrial Revolution, began^2^. Climate change has become an accepted fact that in a variety of ways affects human societies, agriculture, the ecosystem, and the environment. It is associated with changes in the temporal and spatial patterns of main meteorological variables, such as temperature and precipitation^3^. The latest projection of temperature under Shared Socioeconomic Pathways (SSP) scenarios from Coupled Model Intercomparison Project Phase-6 (CMIP6) indicates that the global average temperature will increase by over 5.4 °C in the highest emission scenario and 1.1 °C in the highest mitigation scenario by the end of the twenty-first century^4^. Changes in temperature and precipitation significantly influence various hydroclimatic phenomena, such as droughts and floods. In Poland, similarly, as in many other regions in the world, climate change increases the frequency and intensity of droughts and floods^5^. In recent years, Poland has experienced a greater number of prolonged droughts, heavy rains, and storms. These changes in weather patterns, exacerbated by climate change, also increase the risk of crop failures, wildfires, and water-quality issues^6^. Therefore, a reliable and up-to-date projection of those variables, including local and regional scales, is crucial and urgently needed.

Historical and observed climate change in Poland has been investigated quite often in recent years. Ustrnul et al.^7^ assessed the air temperature changes in Poland for the 1951–2018 period. The assessment of air temperature changes revealed significant trends linked to rising temperatures. The results indicated that average annual and seasonal air temperature and the number of hot days experienced an increasing trend, while the number of frost days decreased. Kejna and Rudzki^8^, in a comprehensive study, evaluated the spatial distribution of air temperature variability in Poland from 1961 to 2018. The authors concluded that average air temperature has increased by 0.33 °C per decade. A significant increase of more than 0.4 °C per decade was observed in the Western part of Poland and Baltic Coast regions. In addition, the increase in July (0.48 °C), January (0.46 °C), and April (0.41 °C) were more than other months for summer, winter, and spring, respectively, while there was no significant increase in air temperature in autumn. The changes in observed sums of precipitation in Poland for 1961–1990 and 1991–2017 periods were investigated by Pińskwar et al.^9^. They found a significant increasing trend for annual and spring precipitation. Also, the monthly precipitation increased for February, March, July, September, October, while it decreased for June, August, November, and December.

In addition, previous studies have projected future climate conditions in Poland under climate scenarios from CMIP5. In general, it is projected that, by the end of the twenty-first century, air temperature in Poland will increase by 1–5 °C under Representative Concentration Pathway (RCP) scenarios^10,11^. Also, the precipitation totals will increase for most stations in Poland for the future period from CMIP5 simulations. It is expected that precipitation in Poland will increase by 5% (minimum), and 16% (maximum) under RCP4.5 and RCP5–8.5 scenarios^6,10–13^.

On the global scale, there is a huge number of studies analyzing climate change and its impact. Furthermore, many of them have projected temperature and precipitation change under climate-change scenarios^14–19^.

General circulation models (GCMs) are the most common tools for gaining quantitative understanding of climate change impacts on regional and global scales. These models are known as the most valuable and effective methods to evaluate the impact of climate change on hydrometeorological events^20,21^.

Currently, CMIP6 GCMs output developed by various institutions in the world have become widely available. It is expected that CMIP6 models, owing to their significant and higher vertical resolution, revised microphysics parameterizations, and modified ocean-ice models, provide better performance than CMIP5^22,23^. In CMIP6, SSP scenarios replaced the RCP scenarios utilized in CMIP5 for future projections^24^. SSP scenarios are used to estimate emission scenarios by taking into consideration potential future changes in socio-economic conditions, including those affecting ecosystems, the population, resources, and institutions^25,26^.

The existing studies evaluating climate change impact in Poland for a future period have mainly focused on the projection of main climate variables i.e., temperature and precipitation. Additionally, previous studies evaluated the performance of CMIP5 models under RCP scenarios. Accordingly, because the CMIP6 models will improve the projection and estimation of future climate, more detailed assessments are required for future changes in air temperature, precipitation and their extreme values under the new SSP scenarios in Poland.

According to the literature review, although the projected impacts of global climate change on temperature and precipitation have significant effects on human life, to the best knowledge of the authors, this is the first attempt to employ the new SSP scenarios from CMIP6 to evaluate climate change in Poland and more specifically to estimate occurrences of extreme temperature and precipitation in the study area. Therefore, this research aims to evaluate the capability of GCMs from CMIP6 in projecting future climate in Poland and projecting the main meteorological variables and extreme climate variables in Central Poland (Toruń) as a case study for a future period under SSP scenarios. Consequently, projected future temperature and precipitation will assist the scientific community in leveraging CMIP6 data to project temperature and precipitation in Poland and similar climate zones. Since understanding future temperature conditions will have a direct impact on human society, agricultural development and water accessibility, the research outputs will provide significant information for policymakers to make socio-economic adjustments for potential critical issues in the future.

It is worth mentioning that, to date, there are no regional climate models (RCMs) publicly available for the daily dataset from CMIP6. Even though most previous studies assessed the capability of GCMs to project meteorological parameters, we used a new high-resolution dataset (0.25° × 0.25°) from NASA Earth Exchange Global Daily Downscaled Projections (NASA NEX-GDDP), in contrast with previous studies that used coarse-resolution (e.g., 2.5° × 2.5°, 1° × 1° and 0.5° × 0.5°) climate data. Additionally, previous studies showed that the NEX-GDDP dataset presents performance superior to that of raw GCMs and even RCM models such as CORDEX in projecting daily precipitation and temperature for the future period^27–30^.

### Study area, datasets and methods

**Figure 1:**
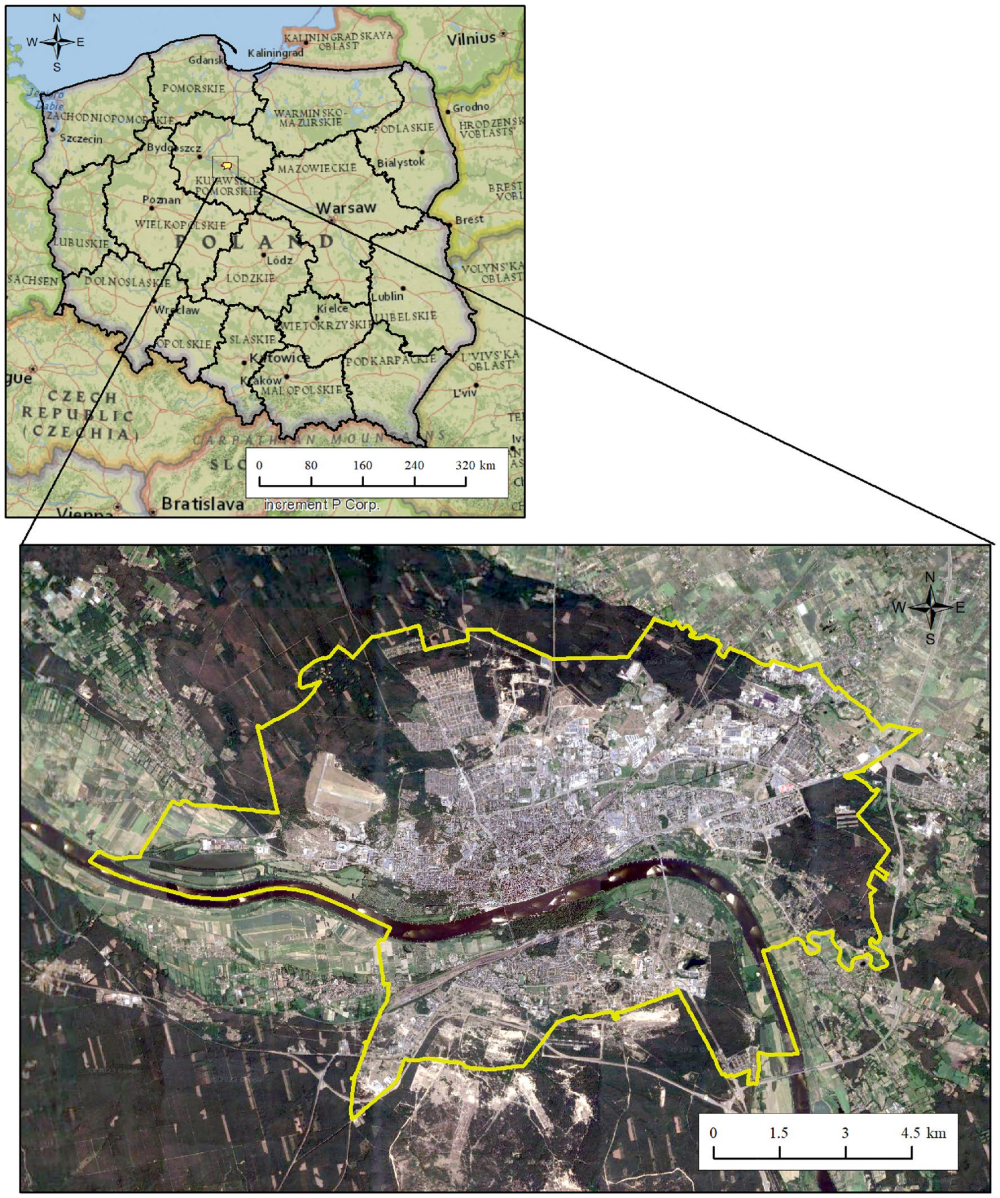
Geographical location of the study area in Poland created using ArcMap 10.8.1 (http://www.esri.com/).

In this research, a region encompassing Toruń lying in the Vistula River basins in north-central Poland (Fig. 1) was chosen as a case study to project the possible impacts of climate change on air temperature, precipitation, and their extreme values. The area of Toruń (53° 2ʹ N, 18° 35ʹ E) is 115.7 km^2^, with an average elevation of 65 m a.s.l.^31^. The mean annual temperature in the period 1950–2022 was 8.3 °C, which was 0.4% higher than Poland’s average temperature. The mean annual sum of precipitation in the area was 538 mm. The study area has a marine west coast, warm summer climate (Cfb) based on Köppen–Geiger climate classification.

### Dataset

The dataset for daily meteorological variables for Toruń, Poland from 1990 to 2014 was obtained from the Institute of Meteorology and Water Management—National Research Institute (IMGW-PIB) (https://www.imgw.pl, data access, December 2022). The daily temperature and precipitation dataset for GCMs was extracted from the NASA Center for Climate Simulation website (https://registry.opendata.aws/nex-gddp-cmip6/, data access, November 2022). In the GCM models, the dataset for the historical reference period is from 1950 to 2014, and the dataset for future climate scenarios is from 2015 to 2100. In this research, we selected equal 25-year periods for the historical reference period (1990–2014), the near-future (2026–2050), mid-term (2051–2075) and far-future (2076–2100) periods. The selected models covered the required variables for this study’s purposes (precipitation, air temperature, maximum and minimum air temperature).

## Methods

The GCMs are reliable tools developed to evaluate changes in meteorological variables, accounting for the impact of climate change under future scenarios^17^. On a global scale, GCMs provide estimates of climate variables (e.g., air temperature, precipitation) based on physical processes occurring in the atmosphere, ocean, cryosphere, and land surface^32^. However, there are considerable uncertainties in the projections of future climate by GCMs. Thus, the output of such a single GCM is not reliable to evaluate future climate. As a result, an ensemble of several GCMs can be used for a reliable projection of future climate variables. Consequently, in this research, an average mean ensemble of several GCMs from CMIP6 is used. The concept of an average mean ensemble of GCMs in addressing the uncertainty of projection of future climate conditions has been approved in various studies^33–35^.

These GCMs are from the NASA Earth Exchange Global Daily Downscaled Projections (NEX-GDDP-CMIP6) dataset based on CMIP6. Table 1 presents the detailed features of GCMs used in this study. GCM models are under two climate-change scenarios from CMIP6, namely SSP1–2.6 and SSP5–8.5. The SSP5–8.5 scenario is characterized by a fossil-fuel-based and energy-intensive economy, with a projection of an increase in mean global temperature of 4.4 °C compared to pre-industrial levels by the end of the century. This scenario is classified as a high-emission scenario and is regarded as a baseline scenario with very high emissions, assuming no implementation of policies. In contrast, the SSP1–2.6 scenario is considered a low-emission scenario, and it is projected that the mean global temperature under this scenario will reach 1.8 °C above pre-industrial levels. The NEX-GDDP CMIP6 dataset provides a downscaled daily precipitation, mean temperature, minimum and maximum temperature data for historical and future periods. These datasets have a spatial resolution of 0.25° and covers the period from 1950 to 2100 (the 1950–2014 historical reference period and 2015–2100 for future periods). This dataset is preferred over others due to its better spatial and temporal resolution. The NEX-GDDP dataset has been widely and successfully used in the latest climate-change studies^36–38^.

**Table 1:** Detailed features of the GCMs used in this study.

GCM model	Research center	Resolution
CMCC-ESM2	The Euro-Mediterranean Centre on Climate Change/Italy	0.25 × 0.25
EC-Earth3	EC-EARTH European consortium/Europe	0.25 × 0.25
INM-CM4-8	Institute for Numerical Mathematics/Russia	0.25 × 0.25
IPSL-CM6A-LR	Institute Pierre-Simon Laplace/France	0.25 × 0.25
MPI-ESM1-2-HR	Max Planck Institute for Meteorology/Germany	0.25 × 0.25
NorESM2-LM	Norwegian Climate Centre/Norway	0.25 × 0.25

In order to conduct this research, we followed three main stages:

1. Projection of annual and seasonal changes in main climate variables (i.e., air temperature and precipitation).
2. Estimation of change in extreme climate variables for future period under scenarios SSP1–2.6 and SSP5–8.5.
3. Comparing the result for projected precipitation, air temperature and extreme values (Table 2) from the historical reference period (1990–2014) with near-future (2026–2050), mid-term (2051–2075), and far-future (2076–2100) in Central Poland.

**Table 2:** Selected extreme climate indices used in this study and their criteria.

No.	Index name	Description
1	Hot days	Days with Tmax > 25 °C
2	Very hot days	Days with Tmax > 30 °C
3	Extremely hot days	Days with Tmax > 35 °C
4	Slight frost days	Days with Tmin < 0 °C
5	Frost days	Days with Tmax < 0 °C
6	Severe frost days	Days with Tmax < ˗10 °C
7	Tropical nights	Days with Tmin > 20 °C
8	Moderately heavy precipitation days	Days with precipitation > 10 mm
9	Heavy precipitation days	Days with precipitation > 20 mm
10	Very heavy precipitation days	Days with precipitation > 30 mm

Although the employed GCM models are downscaled to a higher resolution, due to significant biases with observation data, these models cannot be used directly to evaluate climate systems. To compute regional climates from GCMs, using bias-correction method is crucial. This research applies one of the most common and robust bias-correction methods, i.e. quantile mapping (QM)^39–41^. In the QM methods, all statistical moments of GCM outputs are matched with the observation dataset. In this method, the GCM data are adapted to observed data by using cumulative distribution functions (CDFs)^42^. For future climate projections, CDFs are derived from CDFs linked to GCM outputs in the historical period and take into account future GCM scenarios. Finally, the CDFs of observed variables can be used to estimate corrected values for future periods. The detailed information related to the QM can be found in the literature^43–46^. The capability of the QM bias-correction method has been approved in previous studies^47–51^. To derive the bias correction, a long-term dataset is required to reduce biases in determining bias correction. Therefore, 25-years observed and simulated daily data were used.

In this study, the climate extremes were defined using the set of thermal and precipitation indices listed in Table 2 together with the utilized criteria. These extreme climate indices have been used in various studies in the Polish literature^52,53^.

### Evaluation criteria

To evaluate the performance and capability of climate models, GCMs should be compared with observation data. Climate model performance can be evaluated using several criteria^54^. In this research, the performance of models was evaluated based on coefficient of determination (R-square) and root mean square error (RMSE). The equations for calculating R-square and RMSE are depicted in Eqs. (1) and (2). The best model performance between model data and observed data is R^2^ = 1, and RMSE = 0.

1 $$ R^{2} = \left( {\frac{{\left[ {\sum\nolimits_{i = 1}^{n} {(O_{i} - \overline{O} } )(H_{i} - \overline{H} )} \right]}}{{\sqrt {\sum\nolimits_{i = 1}^{n} {(O_{i} - \overline{O} )^{2} } } \sqrt {\sum\nolimits_{i = 1}^{n} {(H_{i} - \overline{H} )^{2} } } }}} \right)^{2} , $$

2 $$ RMSE = \sqrt {\frac{1}{n}\sum\limits_{i}^{n} {(H_{i} } - O_{i} )^{2} } , $$

where *n* is the number of samples, *O*_*i*_, and *H*_*i*_ are observed data and historical model values at the *i* time and $\overline{O }i$ and $\overline{H }i$ are the mean of the observed and model values, respectively.

## Results

**Figure 2:**
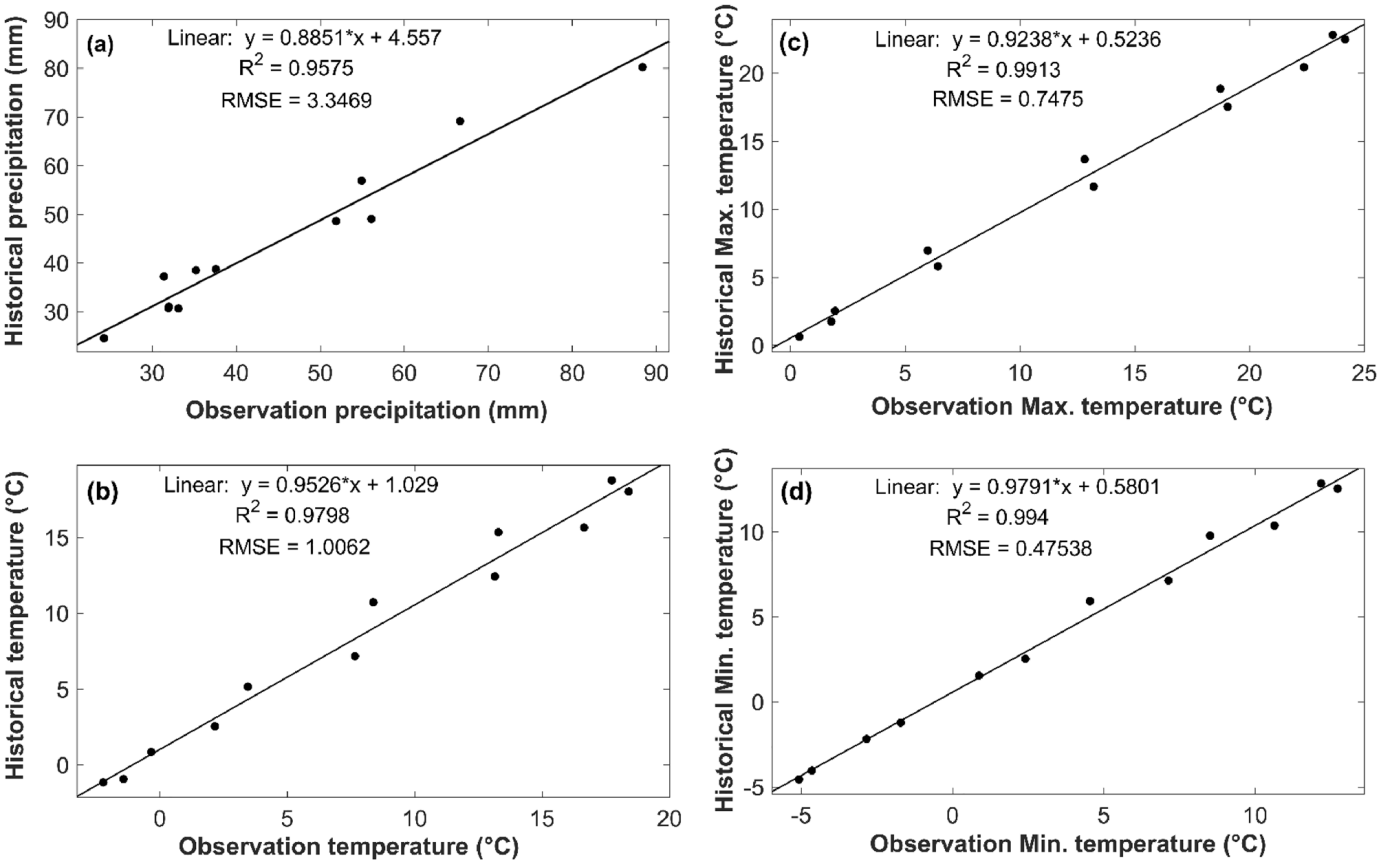
Comparison of observation and bias-corrected historical data in the form of scatter plot, (**a**) precipitation, (**b**) temperature, (**c**) maximum temperature, and (**d**) minimum temperature for the 1990–2014 period.

**Figure 3:**
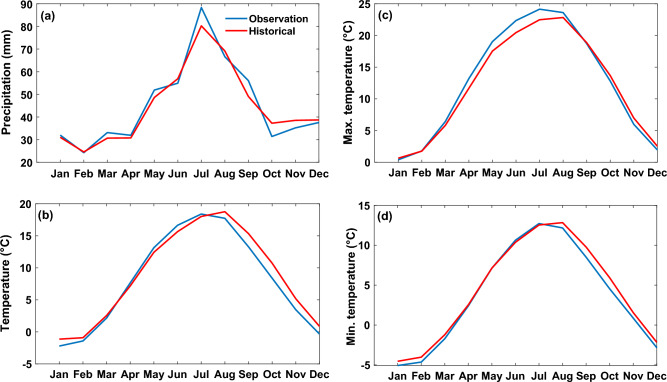
Comparison of seasonal temporal resolution of observation and historical bias-corrected (**a**) precipitation, (**b**) temperature, (**c**) maximum temperature, and (**d**) minimum temperature, for the 1990–2014 period.

Primarily, the bias-correction method was applied to all climate variables to improve the capability of these models and to reduce biases. The results of the ensemble mean of the bias-corrected model in the compassion of observations is depicted in Figs. 2 and 3 as scatterplot form. The comparison of historical data and observation values in Fig. 2 presents the results of R-square and RMSE values. In general, the results showed that there is an acceptable correlation between observation and bias-corrected data.

The results depicted in Figs. 2 and 3 for comparing observed climate variables against the historical GCMs show that the R-square values are in the ranges of 0.95–0.99, RMSE for precipitation is 3.3 mm, and temperature values (mean, min, and max temperature) are in the range of and 0.47–1 °C. These results demonstrated that the selected GCMs ensemble has a strong correspondence with the observation data. Therefore, the average ensemble of models was used to project mean temperature, total precipitation, and their extreme values for future periods.

The projection of climate change impacts on precipitation and temperature is estimated based on a bias-corrected model. The results of projection for precipitation show that, with the exception of a few years, annual precipitation values will increase for both scenarios in comparison with the historical reference period. It is estimated that the annual precipitation total of 545 mm in the historical period will increase to 561 mm (near-future), 567 mm (mid-term) and 578 mm (far-future) under the SSP1–2.6 scenario. Also, the annual precipitation under the SSP5–8.5 scenario will rise to 572 mm, 585 mm, and 571 mm for near-future, mid-term and far-future, respectively.

**Figure 4:**
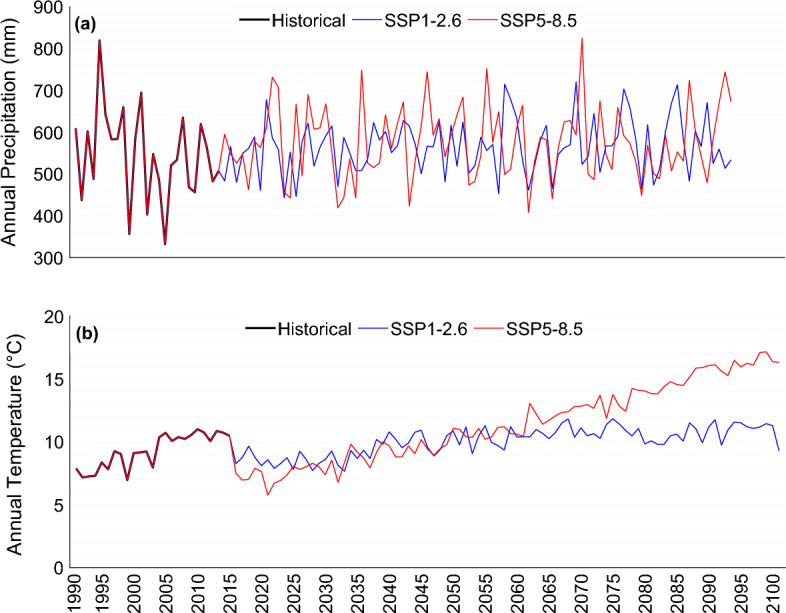
Annual (**a**) precipitation totals and (**b**) temperature changes for historical reference period (1990–2014), and for future period (2015–2100) projected under scenarios SSP1–2.6 and SSP5–8.5.

**Figure 5:**
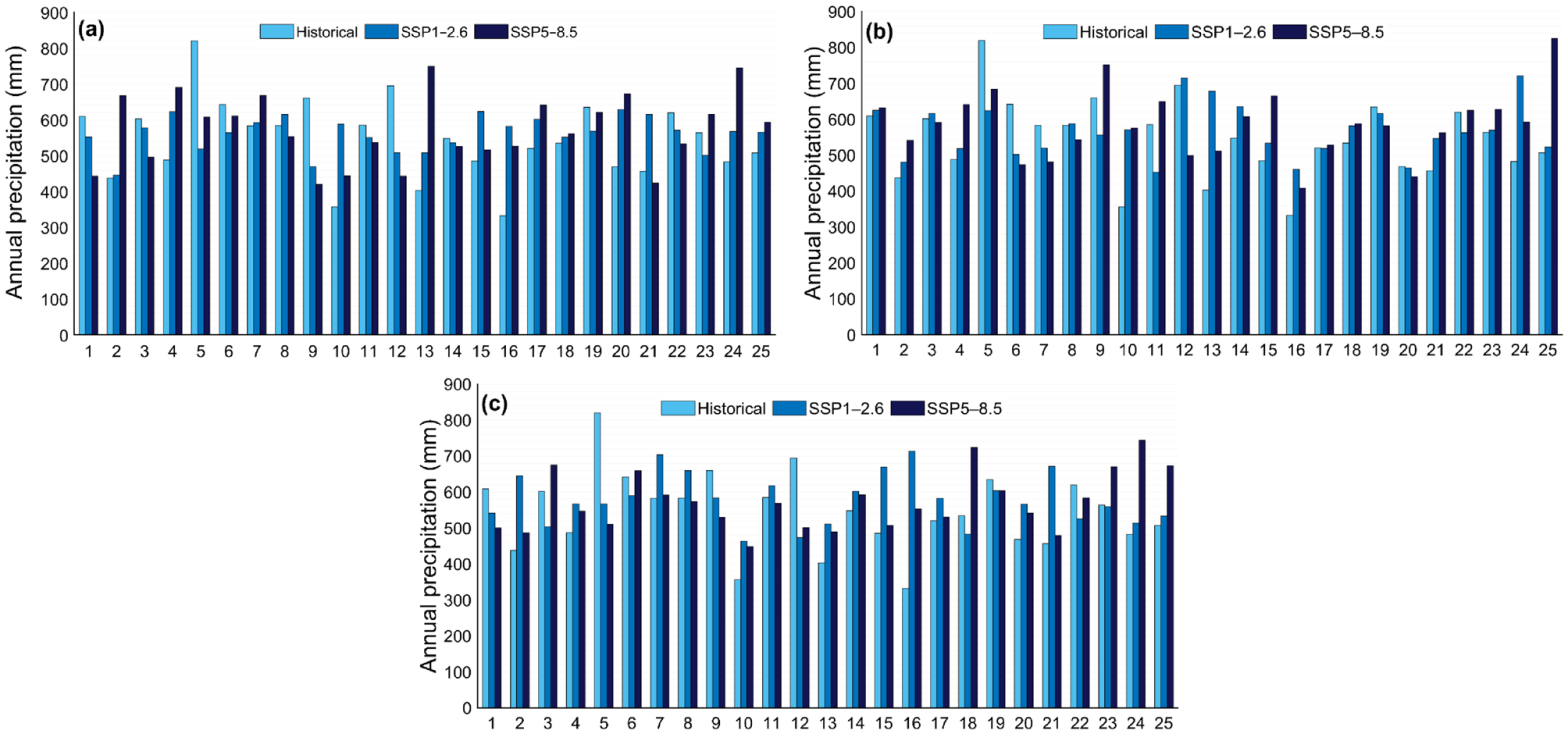
Comparison of annual precipitation totals for historical period (1990–2014) with their projections under scenarios SSP1–2.6 and SSP5–8.5 for (**a**) near-future period (2026–2050), (**b**) mid-term future period (2051–2075) and (**c**) far-future period (2076–2100).

**Figure 6:**
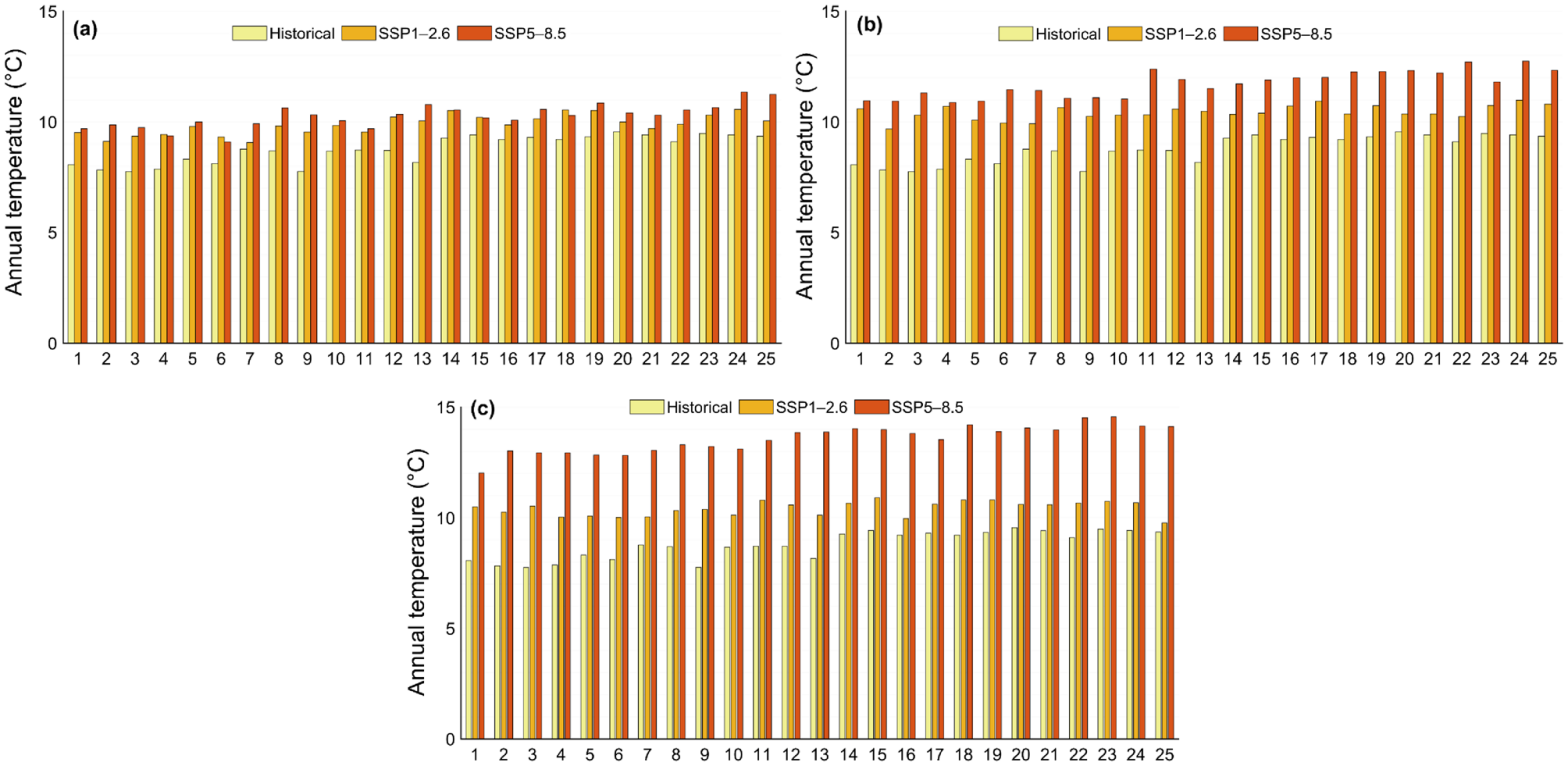
Comparison of mean annual temperature for historical period (1990–2014) with their projections under scenarios SSP1–2.6 and SSP5–8.5 for (**a**) near-future period (2026–2050), (**b**) mid-term future period (2051–2075) and (**c**) far-future period (2076–2100).

**Figure 7:**
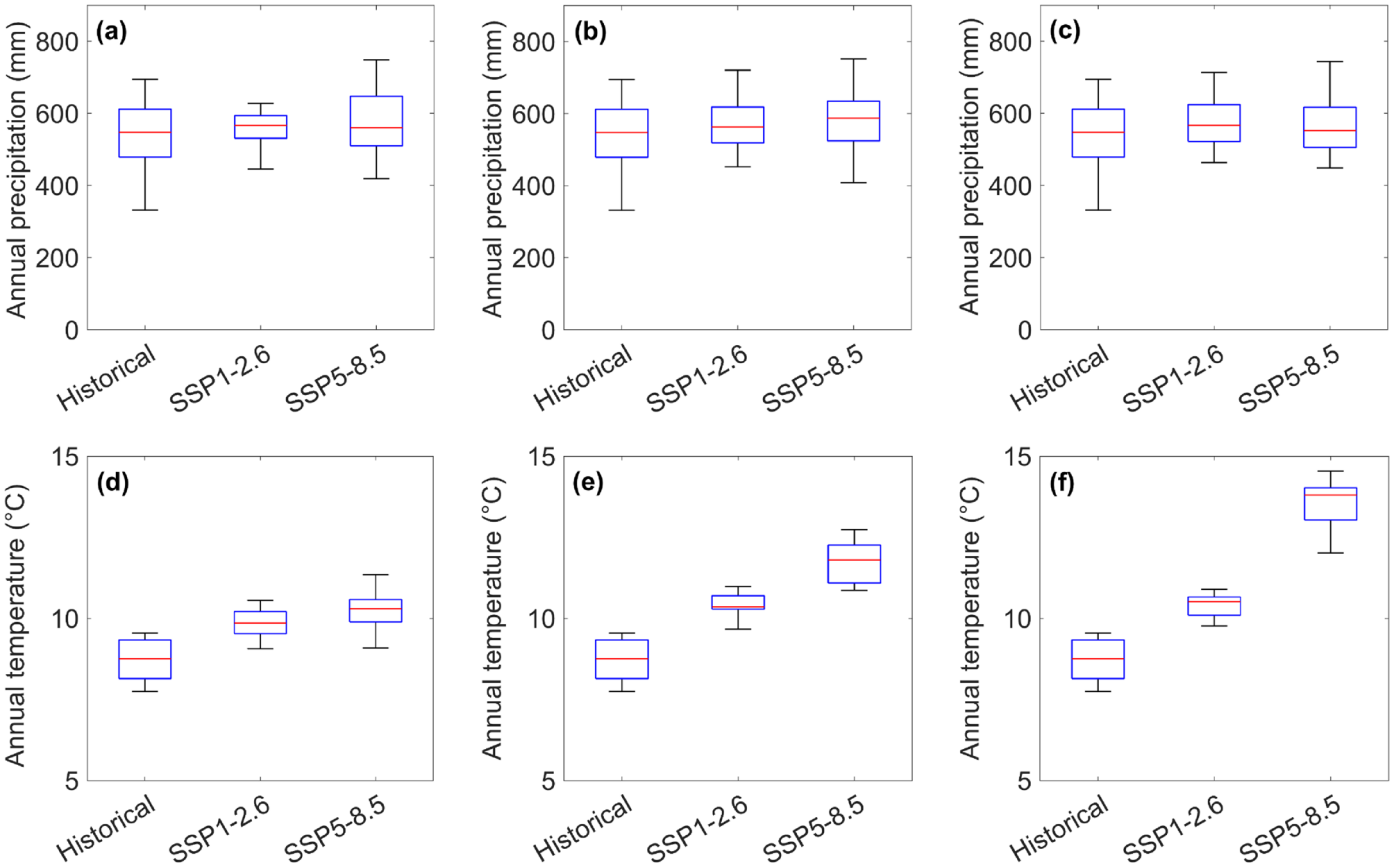
Comparison of mean annual precipitation and temperature for historical period (1990–2014) against their projections under scenarios SSP1–2.6 and SSP5–8.5 for (**a**) precipitation, near-future period (2026–2050), (**b**) precipitation, mid-term future period (2051–2075), (**c**) precipitation, far-future period (2076–2100), (**d**) temperature, near-future period (2026–2050), (**e**) temperature, mid-term future period (2051–2075), and (**f**) temperature, far-future period (2076–2100).

The projected change in mean annual temperature in the study area shows that annual temperature will increase for both scenarios by the end of the twenty-first century. It is expected that the annual temperature of 8.8 °C in the historical reference period will increase to 10.1 °C and 11.5 °C for future periods (2015–2100) under scenarios SSP1–2.6 and SSP5–8.5, respectively. Although the temperature will increase for both scenarios, mean annual temperature for SSP5–8.5 will increase dramatically up to 10.3 °C, 11.7 °C and 13.6 °C, for near-future, mid-term, and far-future periods, respectively. It is also expected that for, SSP1–2.6, the annual temperature will rise to 9.9 °C in near-future, 10.4 °C in mid-term and 10.4 °C for far-future periods. Figure 4 shows the comparison of changes in annual precipitation and temperature for historical (1990–2014) and future periods (2015–2100) under scenarios SSP1–2.6 and SSP5–8.5. Also, the variations in annual precipitation totals and mean annual temperature for every year for historical and future periods are depicted in Figs. 5 and 6, respectively. Figure 7 shows the boxplot form for comparison of mean annual precipitation and temperature for the historical period and future periods.

**Figure 8:**
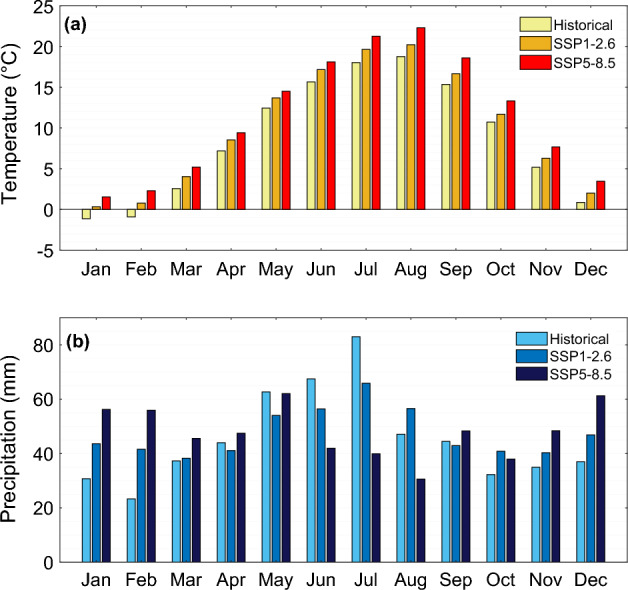
Comparison of (**a**) mean monthly temperature and (**b**) monthly precipitation totals, between historical reference period (1990–2014) and future period (2015–2100) projected under scenarios SSP1–2.6 and SSP5–8.5.

Similar to annual precipitation totals and mean annual temperature, projected changes for months show increases under both climate-change scenarios, with the exception of precipitation from May to August (Fig. 8). The temperature in winter (DJF) will change from − 0.4 °C in the historical reference period to 1 °C and 2.4 °C under scenarios SSP1–2.6 and SSP5–8.5, respectively (Fig. S1a). In the spring (MAM), the temperature will rise from 7.4 °C in the reference period to 8.8 °C under SSP1–2.6 and 9.7 °C under SSP5–8.5. It is projected that the temperature for summer (JJA) and autumn (SON) will also experience an increase. The temperature for the study area during the summer will rise from 17.5 to 19.0 °C and 20.5 °C under the SSP1–2.6 and SSP5–8.5 scenarios, respectively. In autumn (SON), the temperature will reach 11.5 °C in SSP1–2.6 and 13.2 °C SSP5–8.5 in comparison with 10.4 °C in the historical reference period.

The precipitation values in the winter season (DJF) will rise from 91 to 132 mm under SSP1–2.6 and 174 mm under SSP5–8.5 (Fig. S1b). In the spring (MAM), the precipitation will decrease from 144 to 133 mm for SSP1–2.6 and will rise to 155 mm for SSP5–8.5. The results showed that the precipitation will experience a decrease during the summer (JJA) for the study area. It is expected that the mean precipitation total in summer (198 mm in the historical period) will decline to 179 mm under SSP1–2.6 and 113 mm under SSP5–8.5. The precipitation in the study area in autumn (SON) will change from 112 mm in the historical period to 124 mm under SSP1–2.6 and 135 mm for SSP5–8.5 (Fig. S1b).

The estimations of future changes in extreme climate are depicted in Tables S1–S3. In general, the results indicate that the number of days for moderately heavy precipitation (> 10 mm), heavy precipitation (> 20 mm) and very heavy precipitation (> 30 mm) will increase by the end of the twenty-first century. It is projected that in the near-future period the average number of days with moderately heavy precipitation will slightly increase from 13.9 to 15.5 and 15.2 days under SSP1–2.6 and SSP5–8.5, respectively. Also, the number of days with very heavy precipitation will slightly increase from 1.2 to 1.4 and 1.3 for SSP1–2.6 and SSP5–5.8 scenarios, respectively.

In contrast, the average number of days with heavy precipitation in the near-future period will decline negligibly from 4 days in the historical period to 3.9 and 3.8 for scenarios SSP1–2.6 and SSP5–8.5, respectively. Projections of extreme daily precipitation for 2076–2100 are only slightly greater than for 2026–2050, except for the category of very heavy precipitation, for which a strong decline is expected (see Table S1).

The projections of changes in maximum temperature (Tmax) depicted in Table S2 shows that by the end of the twenty-first century, the average number of hot days (Tmax > 25 °C), very hot days (Tmax > 30 °C), and extremely hot days (Tmax > 35 °C) will increase for both scenarios in mid-term and far-future periods. It is expected that, in the near-future period, the average number of those days will decline negligibly under SSP5–8.5 in comparison with the historical period. In contrast, during the near-future period the average number of frost days and very frost days will increase or will be similar under SSP5–8.5 in comparison with the historical period. The results indicated that, in the far-future period, the average number of hot days, very hot days, and extreme hot days will increase significantly (see Table S2). It is estimated that the average number of hot days will increase from 54.6 in the historical period to 70.0 and 128.7 days under SSP1–2.6 and SSP5–8.5, respectively, in the far-future period. The change in the average number of very hot days and extreme hot days is projected to be even more dramatic. It is projected that the average number of very hot days, which stood at 12.3 days in the historical period, will reach 75.5 days under SSP5–8.5 in the far-future period. Additionally, the average number of days for extreme hot days will reach 25.4 days under SSP5–8.5 in the far-future period in comparison with only 1.1 days for the historical period. Also, the results show that the nighttime temperature will significantly change. For instance, the average number of tropical nights (Tmin > 20 °C) will increase to 44.0 under scenario SSP5–8.5 (far-future) in comparison with 1.5 days in the historical period.

The average number of frost days (Tmax < 0 °C) and very frost days (Tmax < − 10 °C) will decline under SSP1–2.6 and SSP5–8.5 scenarios for mid-term and far-future periods. The results reveal that in the near-future period the average number of slight frost days (Tmin < 0 °C) will increase, while in the mid-term and far-future periods it will decrease. The number of those days will decrease dramatically from 92.32 in the historical period to 14.8 under scenario SSP5–8.5 in the far-future period.

The demonstration of results in the monthly resolution for all extreme climate variables illustrated in Figs. S2 to S4 shows that the average number of days for extreme climate, even at monthly resolution, follows the same trends as mentioned in the annual average number of days for extreme climate.

The results are comprehensively summarized in Table 3.

**Table 3:** Projected changes in mean annual temperature, and annual precipitation total, and their extremes.

Indices name	SSP1–2.6			SSP5–8.5		
	(Near-future)	(Mid-term)	(Far-future)	(Near-future)	(Mid-term)	(Far-future)
Temperature	1.10 °C	1.65 °C	1.64 °C	1.48 °C	2.95 °C	4.79 °C
Precipitation	16 mm	22.75 mm	33.48 mm	27.20 mm	40.60 mm	26.78 mm
Hot days	5.96	18.84	15.64	** − 2.8**	35.16	74.12
Very hot days	3	9.76	6.32	** − 3.08**	22.44	63.2
Extremely hot days	0.32	0.84	0.56	** − 0.72**	5.12	24.28
Slight frost days	9.96	** − 18.28**	** − 14.12**	14.48	** − 28.08**	** − 77.52**
Frost days	6.28	** − 10.8**	** − 13.72**	5.56	** − 19.08**	** − 27.04**
Severe frost days	** − 0.92**	** − 0.96**	** − 1.12**	** − 0.44**	** − 1.12**	** − 1.12**
Tropical nights	0.64	2.4	** − 1.48**	** − 3.12**	7.68	42.48
Moderately heavy precipitation days	1.6	0.92	2	1.28	1.08	1.68
Heavy precipitation days	** − 0.08**	+ 0.4	0.16	** − 0.24**	0.36	0.04
Very heavy precipitation days	0.24	0.4	0.28	0.16	0.2	** − 0.32**

Bold font indicates a decrease in average number of days, while normal font indicates increase in average number of days and projected values.

## Discussion

To date, no regional climate models are available on daily temporal resolutions for the study area. Therefore, GCMs are still the most effective models for evaluating future climate conditions in this region. Additionally, employed GCMs have a high resolution, and the capability and superiority of these models over raw GCMs were approved in previous studies.

Based on a literature review, the existing studies presenting projections of future climate in Poland^6,10,12,55^ are focused on main climate variables and from the CMIP5 simulation. However, to date, there is no study investigating the impact of climate change in Poland through the CMIP6 models and projecting their extreme values for the future period. Most previous projections of future climate change in Poland were conducted using CMIP5 models under RCP scenarios. Previous studies for some areas in Europe and the globe show the capability of state-of-the-art CMIP6 data in the better projection of future climate condition on a global scale^23,56–58^. Also, these studies confirm that there are differences in the projected change for climate conditions for CMIP5 and CMIP6 models. Therefore, to evaluate the differences between the results of various models (CMIP5 and CMIP6), our results were compared with other results available in the literature.

The projected change in mean annual air temperature for this study (1–4.8 °C) for the two analyzed scenarios shows that there are differences for projected values from CMIP5 and CMIP6. Mezghani et al.^13^ projected changes in temperature under RCP4.5 and RCP8.5 scenarios from CMIP5. The projected results indicated that the annual temperature for Poland will increase by 1 °C and ~ 2 °C for near-future (2021–2050) and far-future (2071–2100) periods, respectively, under the RCP4.5 scenario. The authors stated that, by the period 2071–2100, the annual temperature is projected to rise by 4 °C under the RCP8.5 scenario. Piniewski et al.^12^ estimated future changes in temperature for the Vistula and Oder river basins. The results were partly close to those conducted by Mezghani et al.^13^. The projected estimations showed that the temperature for study area will rise by 1–1.5 °C in the near-future period and by 1.8–3.7 °C in the far-future in relation to mean from the reference period. Szwed^10^ concluded that the average annual temperature would increase in the future for Poland. It is projected that, in the near future (2021–2050), the temperature increase will be 1 °C for RCP4.5 and 1.5 °C for the RCP8.5. For a far future period (2071–2100), the average annual temperature will increase by 2 °C under RCP4.5, while for RCP8.5 the temperature increase will range between 3 and 4 °C in the west and north-east of Poland, respectively.

The differences between the present study results and previous studies are mainly caused by differences between the CMIP5 and CMIP6 models. Also, these results confirm the previous studies that stated the air temperature will increase under CMIP6 scenarios like in CMIP5 scenarios. However, the increases in air temperature under CMIP6 would be greater than under CMIP5 scenarios, in some parts of the world. For example, Palmer et al.^57^ compared the performance of CMIP6 and CMIP5 projection for main climate variables (temperature and precipitation) in central Europe, northern Europe, and the Mediterranean. The results showed that the projected increase in mean summer warming from CMIP6 models would be significantly stronger in comparison with CMIP5 models, particularly in central Europe. Cos et al.^58^ investigated the impact of climate change on Mediterranean. The authors concluded that temperature increases for the summer seasons in CMIP6 in the range of 1.83 to 8.49 °C would be greater than CMIP5 future scenarios with 1.22 to 6.63 °C. Moreover, on global scale, the mean annual temperature under SSP scenarios from CMIP6 would be greater than under RCP scenarios from CMIP5^23,24,56^.

In this study, the annual precipitation totals are projected to increase by 2–6% under SSP1–2.6 scenarios and 4–7.5% under SSP5–8.5. The previous studies projected that the precipitation will increase by 5–16% for Poland under scenarios RCP4.5 and RCP5-8.5^6,10–13^. The differences between the present study results and previous studies for precipitation are partly related to the selection of GCM models, future scenarios, bias-correction methods, and time horizon. However, previous studies also confirms the superiority of the CMIP6 models over CMIP5 in precipitation projections. Khadka et al.^59^ evaluated the CMIP5 and CMIP6 models for simulation of future precipitation in Southeast Asian monsoon domain. The results indicated that CMIP6 models show better representation of annual rainfall cycles and spatial pattern than CMIP5. The authors concluded that there was a significant improvement in rainfall and large-scale circulation simulations by CMIP6 models over CMIP5 models, which may have been attributed to CMIP6 models’ higher spatial resolutions, increased vertical levels, better parameterization of the atmosphere and land surface, etc.

Although the precipitation was expected to increase for both scenarios, the seasonal precipitation in the summer showed a decrease. We reviewed the literature to justify these unexpected results. It is indicated that the same trends were also experienced in previous studies for seasonal projections of precipitation. For example, Mezghani et al.^11^ based on the empirical–statistical downscaling (ESD) method concluded that the precipitation in summer and autumn will decrease for 2021–2050 and 2071–2100 periods under RCP8.5 scenario. The results of Kundzewicz et al.^6^ showed that the projected precipitation trends show uncertainties for seasonal changes. They found that precipitation will increase significantly for winter and spring under all RCP scenarios, no statistically significant changes in precipitation were expected in summer and autumn. Mezghani et al.^55^ also mentioned the same uncertainties for seasonal changes in the projected precipitation. The authors concluded that the precipitation range in summer (**− **5 to 9%) and autumn (**− **4 and 13%) shows a disagreement with projected precipitation for future periods.

In general, the change in extreme indices has good correlation with previous studies. The results revealed that the 25-year average number of hot days, very hot days, and extreme hot days will increase by between 3 and 63 days. Szwed^10^ also mentioned that the average number of extreme temperature days (Tmax > 30 °C) will increase by 3–4 days in RCP4.5 and even by several weeks under RCP8.5 and the far-future period. The average number of frosty days (Tmin < 0 °C) is expected to decrease even by 27 days under the SSP5–8.5 scenario and far-future period according to this study, while it is expected to decrease by even more than 10 days on average by the study conducted by Szwed^10^. The only unexpected results were the decreasing the number of hot days and increasing number of frost days for near-future period under the SSP5–8.5 scenario (Tables S2, S3). In general, there is good agreement between our results and previous studies that confirm that the number of hot and frosty days will, respectively, increase and decrease under RCP8.5 scenarios from the CMIP5 simulation for the far-future period^10,60^. However, the number of hot days in the near-future period in this research will slightly decrease and the number of frosty days increased in the same period and scenario. Szwed^10^ also mentioned that the number of hot days and frosty days will experience only a minor change in the near-future in Poland. Therefore, it seems that the slight differences between the results of this study and the results expected based on previous studies are partly related to the uncertainties of climate models and scenarios.

According to Pińskwar and Choryński^61^ projection, heavy precipitation (> 10 mm) in Poland will increase for both seasonal and annual resolution under the RCP4.5 and RCP8.5 scenarios. These results are in good correspondence with our results, which also show an increase in the average number of days with extreme precipitation.

In summary, there is no doubt that the projections of future climate conditions include various uncertainties^62,63^. This fact is also confirmed in the cited literature for Poland. The choice of model simulation, future climate scenarios, bias correction, and downscaling methods are the main sources of uncertainties^64^. In this regard, for future climate studies, researchers have preferred “projections” over “predictions”^65^. Therefore, in light of the emphasized sources of uncertainties for projections of future climate conditions, it is to be expected that the results of this study should differ from those of previous studies in some cases.

## Conclusions

This study projected future changes in main climate (i.e., temperature and precipitation) as well as extreme climate variables for Central Poland. An average mean ensemble of six GCM models has been used under two climate-change scenarios—SSP1–2.6 and SSP5–8.5—for three future periods: near-future (2026–2050), mid-term (2051–2075) and far-future (2076–2100). In general, comparison of these results with previous studies shows the capability of bias-corrected GCMs from CMIP6 in projections of future climate in Central Poland.

To conclude, the results indicated that, by the end of the twenty-first century, the temperature in Central Poland will increase by 1–4.8 °C and precipitation by 2–7.5%. The average number of all categories of hot days and days with extreme precipitation will also increase, while the average number of light frosty days, frost days, and severe frost days will decline by the end of the twenty-first century under the SSP1–2.6 and SSP5–8.5 scenarios.

The results of this study have some practical implications for the scientific community in the evaluation of possible impacts of climate change on various extreme hydrological and extreme events such as floods and droughts. Additionally, the results of this research provide precious information for decision-makers to take some measurements and strategies to adapt to and mitigate extreme climatic events in the future.

Building on this research, future research could evaluate the probability of occurrence of the most hazardous extreme hydrological and climate events such as floods and droughts using different CMIP6 models for various climate-change scenarios.

### Supplementary Information

Supplementary Information.

## Data Availability

The datasets for the GCMs from CMIP6 are available at 10.1038/s41597-022-01393-4 (accessed on 22 November 2022) and from the NEX-GDDP-CMIP6 NASA Center for Climate Simulation (10.7917/OFSG3345). The additional data that support the findings of this study are available from the corresponding author upon reasonable request.
