# Supplementary material for: Projection of climate change impacts on extreme temperature and precipitation in Central Poland
Source: Sci Rep. 2023 Oct 31;13:18772. doi: 10.1038/s41598-023-46199-5 (PMC10618218; doi:10.1038/s41598-023-46199-5)
Supplement: Supplementary file 1 — Supplementary Information. [file 41598_2023_46199_MOESM1_ESM.docx]

**Projection of climate change impacts on extreme temperature and precipitation in Central Poland**

Babak Ghazi ^a^, Rajmund Przybylak ^a, b^, Aleksandra Pospieszyńska ^a, b^

^a^ Department of Meteorology and Climatology, Faculty of Earth Sciences and Spatial Management, Nicolaus Copernicus University Toruń

^b^ Centre for Climate Change Research, Nicolaus Copernicus University Toruń

Corresponding author: Babak Ghazi, [babak.ghazi@doktorant.umk.pl](mailto:babak.ghazi@doktorant.umk.pl)


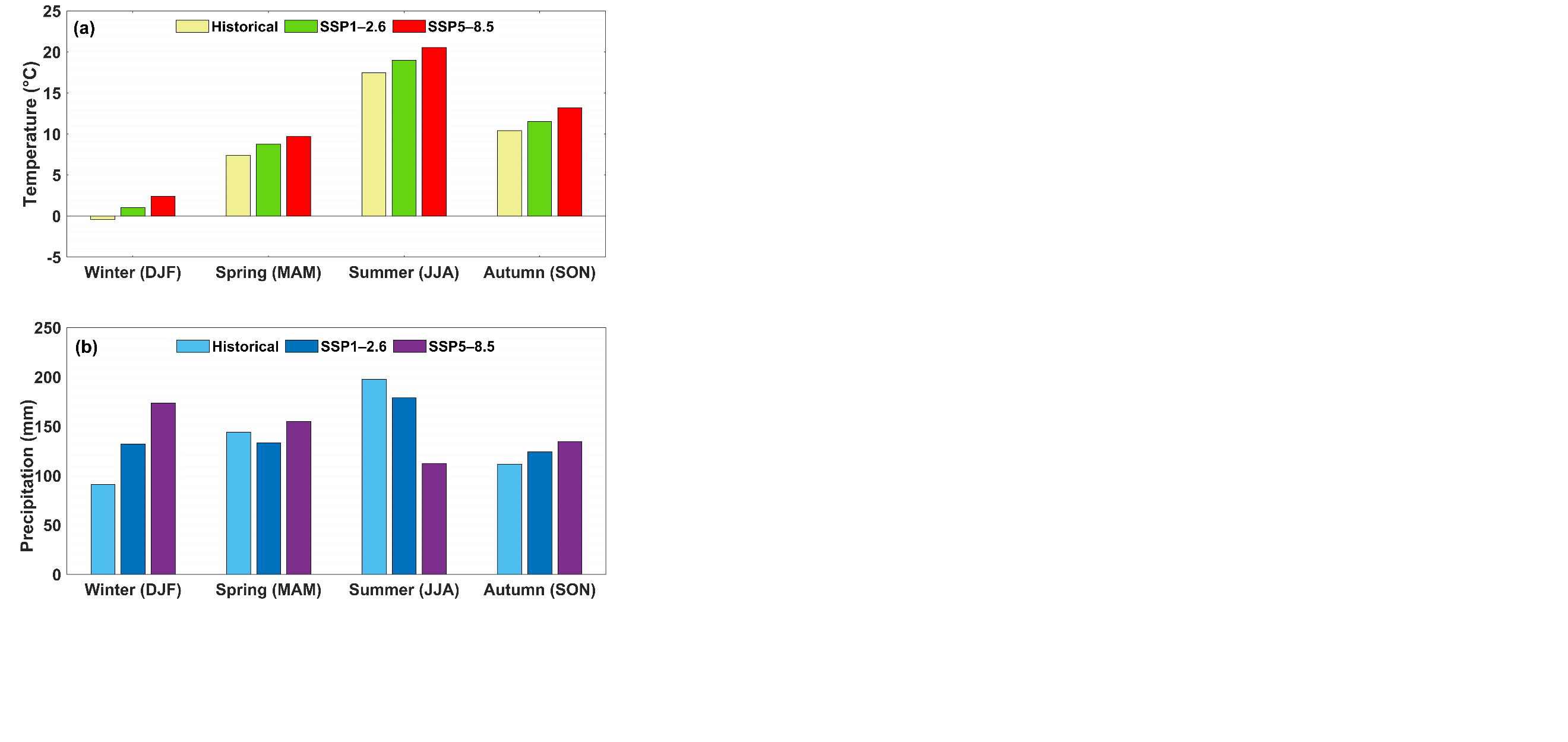


Fig S1. Comparison of seasonal changes in (a) temperature and (b) precipitation for historical and future periods

Table S1. Comparison of average number of days with extreme precipitation for historical and future periods

| Time period | Average number of days with precipitation | | |
| --- | --- | --- | --- |
|  | >10 mm | >20 mm | >30 mm |
| Historical (1990–2014) | 13.9 | 4.0 | 1.2 |
| SSP1-2.6 (2026–50) | 15.5 | 3.9 | 1.4 |
| SSP1-2.6 (2051–75) | 14.8 | 4.0 | 1.6 |
| SSP1-2.6 (2076–2100) | 15.9 | 4.2 | 1.4 |
| SSP5-8.5 (2026–50) | 15.2 | 3.8 | 1.3 |
| SSP5-8.5 (2051–75) | 15.0 | 4.4 | 1.4 |
| SSP5-8.5 (2076–2100) | 15.6 | 4.0 | 0.8 |

Table S2. Comparison of average number of hot days, very hot days, extremely hot days, and tropical nights for historical and future periods

| Time period | Average number of days with temperature | | | |
| --- | --- | --- | --- | --- |
|  | Tmax>25 °C | Tmax>30 °C | Tmax>35 °C | Tmin>20 °C |
| Historical (1990–2014) | 54.6 | 12.3 | 1.1 | 1.5 |
| SSP1-2.6 (2026–50) | 60.5 | 15.3 | 1.4 | 2.1 |
| SSP1-2.6 (2051–75) | 73.4 | 22.0 | 1.9 | 3.9 |
| SSP1-2.6 (2076–2100) | 70.2 | 18.6 | 1.6 | 0.0 |
| SSP5-8.5 (2026–50) | 51.8 | 9.2 | 0.4 | 0.8 |
| SSP5-8.5 (2051–75) | 89.7 | 34.7 | 6.2 | 9.2 |
| SSP5-8.5 (2076–2100) | 128.7 | 75.5 | 25.4 | 44.0 |

Table S3. Comparison of average number of frost days, very frost days, and light frost days for historical and future periods

| Time period | Average number of days for temperature | | |
| --- | --- | --- | --- |
|  | Tmax<0 °C | Tmax<˗10 °C | Tmin<0 °C |
| Historical (1990–2014) | 27.6 | 1.1 | 92.3 |
| SSP1-2.6 (2026–50) | 33.9 | 0.2 | 102.3 |
| SSP1-2.6 (2051–75) | 16.8 | 0.2 | 74.0 |
| SSP1-2.6 (2076–2100) | 13.9 | 0.0 | 78.2 |
| SSP5-8.5 (2026–50) | 33.2 | 0.7 | 107.2 |
| SSP5-8.5 (2051–75 | 8.6 | 0.0 | 64.2 |
| SSP5-8.5 (2076–2100) | 0.6 | 0.0 | 14.8 |


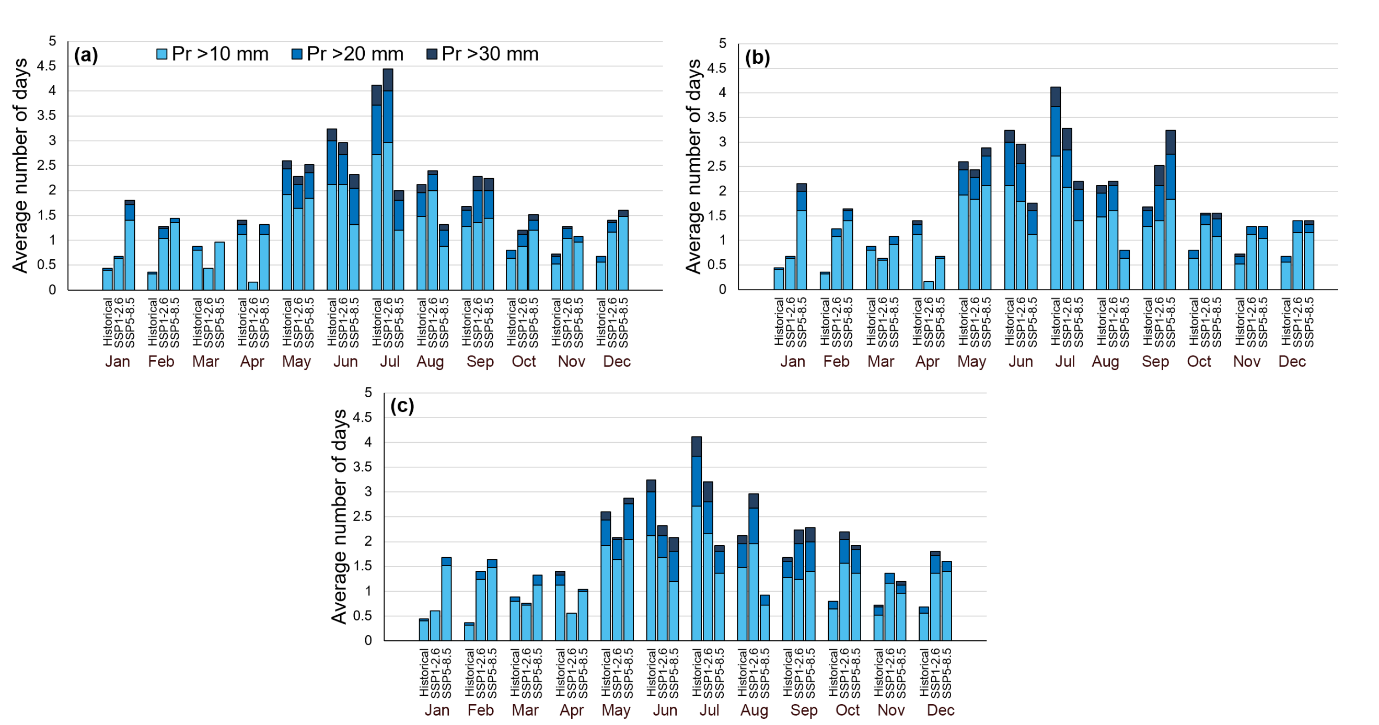


Fig S2. Comparison of average number of days with extreme precipitation in (a) near-future (b) mid-term future, and (c) far-future periods


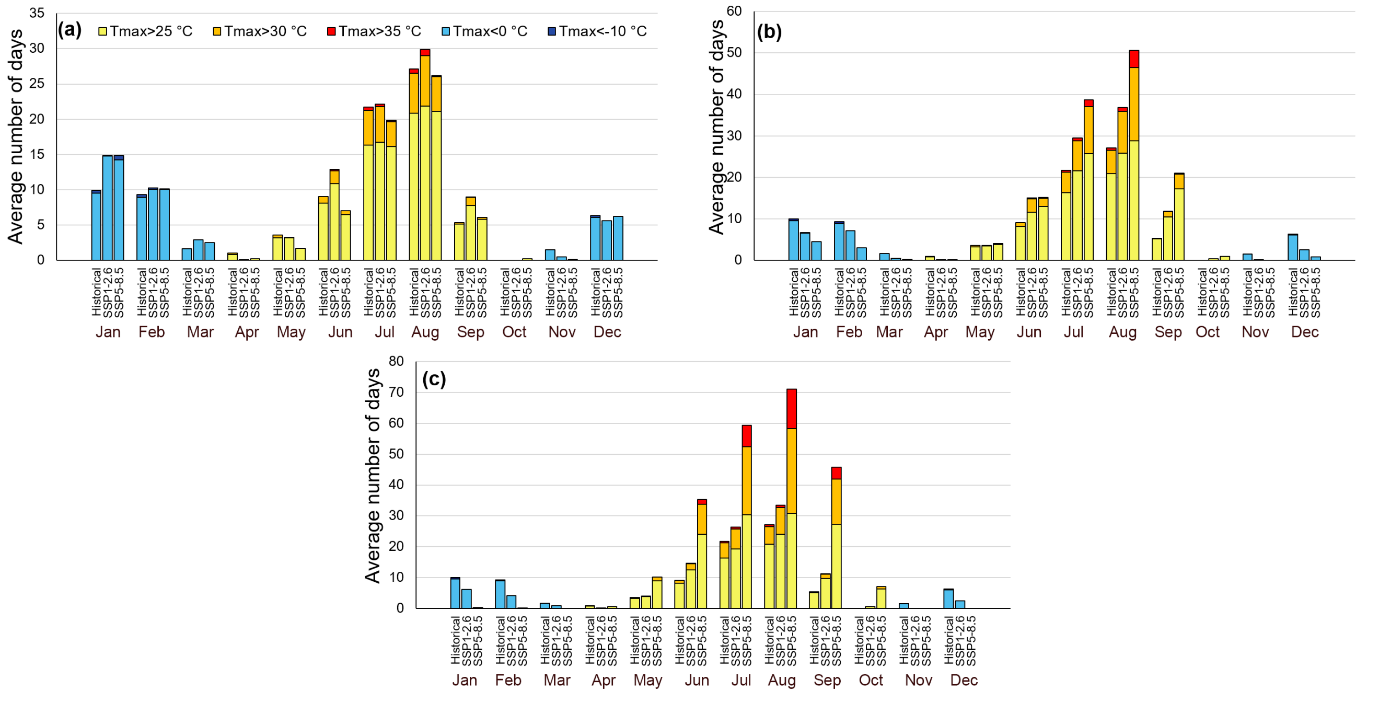


Fig S3. Comparison of average number of days with maximum temperature in (a) near-future (b) mid-term future, and (c) far-future periods


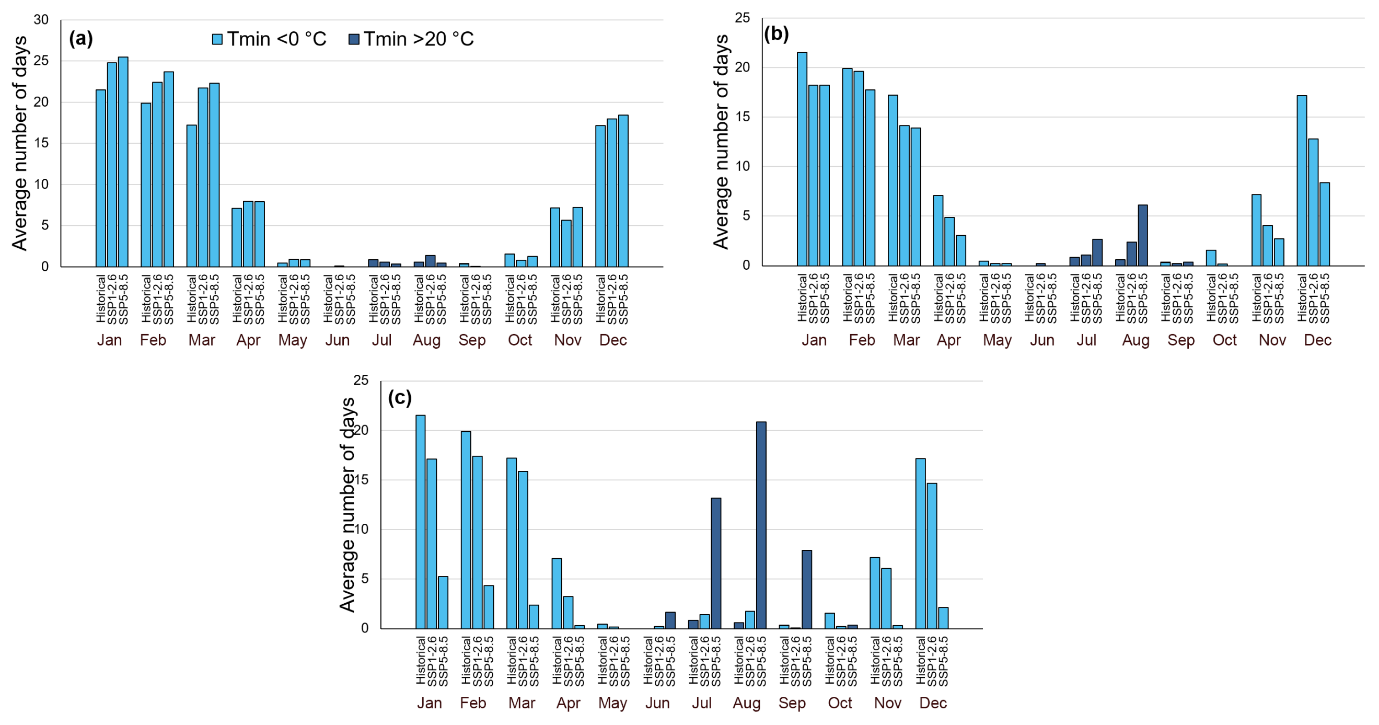


Fig S4. Comparison of average number of days with minimum temperature in (a) near-future (b) mid-term future, and (c) far-future periods
